# Cervical cancer screening “see and treat approach”: real-life uptake after invitation and associated factors at health facilities in Gondar, Northwest Ethiopia

**DOI:** 10.1186/s12885-021-08761-0

**Published:** 2021-09-16

**Authors:** Alemnew Destaw, Miresa Midaksa, Adamu Addissie, Eva Johanna Kantelhardt, Muluken Gizaw

**Affiliations:** 1 Department of epidemiology and Biostatistics, School of public health, College of Medicine and Health Science, Mizan-Tepi University, Mizan-Teferi, Ethiopia; 2 Addis Ababa Food, Medicine and Health Care Administration and Control Authority, Addis Ababa, Ethiopia; 3 Department of Preventive medicine, Addis Ababa University, School of Public Health, Addis Ababa, Ethiopia; 4 Institute for Medical Epidemiology, Biometrics and Informatics Martin-Luther-University, Halle-Wittenberg, Germany; 5 Department of Gynecology, Martin-Luther-University, Halle-Wittenberg, Germany

**Keywords:** Cervical cancer, Screening uptake, VIA approach, Ethiopia

## Abstract

**Background:**

Although cervical cancer is a preventable disease, screening coverage in Ethiopia is far below the target. There is limited evidence on uptake among the general population in Ethiopia. Thus, this study was conducted to assess uptake and associated factors with the cervical cancer screening “see and treat approach” among eligible women in public health facilities in Gondar town, Northwest Ethiopia.

**Method:**

A facility-based, cross-sectional study was conducted. The total sample size was 493. A consecutive sampling method was applied. Participants were informed about and invited to cervical cancer screening using visual inspection with acetic acid. Crude and adjusted odds ratios were calculated to determine statistical association with socio-demographic variables. Multivariable logistic regression was used to determine factors of cervical cancer screening uptake.

**Result:**

Out of 464 women advised for screening, 76 (16.4, 95% CI [13, 19.8%]) attended the screening. Primary education and above (AOR = 5.3, 95% CI [2.20, 13.0]), knowledge about the disease (AOR = 8.4, 95% CI [3.33, 21.21]), perceived susceptibility (AOR = 6.5, 95% CI [2.72, 15.51]), fewer perceived barriers (AOR = 6.4, 95% CI [2.30, 17.80]), cues to action (AOR = 4.6, 95% CI [1.86, 11.32]), perceived self-efficacy (AOR = 5, 95% CI [2.14, 11.73]), and previous recommendation for screening (AOR = 2.7, 95% CI [1.15, 6.51]) were significantly associated with screening uptake.

**Conclusion:**

The actual uptake of screening offered in this study was high relative to only 3% national screening coverage. There is a need to implement active invitation for screening with special focus on less-educated women. Repeated invitation may facilitate future screening uptake.

## Introduction

Globally, cervical cancer is the fourth most commonly diagnosed cancer among females, with an estimated 604,127 new cases and 341,831 deaths in 2020 [1] . The highest incidence rates are documented in Southern, Eastern, and Western Africa as well as South-Central Asia and South America [1]. In Ethiopia, cervical cancer is the second leading cause of cancer deaths among females, with an estimated 7745 new cases and 5338 deaths in 2020 [2]. The high burden of cervical cancer in developing countries is due to a lack of access to screening services and delayed treatment [3]. Poverty, little education, living in rural areas, lack of knowledge about cervical cancer, lack of health care infrastructure, and lack of trained practitioners are the main obstacles for the implementation of cervical cancer screening in developing countries [4]. Although cervical cancer is preventable, about 90% of women in these countries have never had screening even once in their lifetime [5].

The world health organization (WHO) developed a comprehensive approach to prevention and control of cervical cancer to identify opportunities for effective interventions [6]. Simplified “screen and treat” (SAT) approaches using visual inspection with acetic acid (VIA) for secondary prevention of cervical cancer have been the recommended screening method in developing countries where resources are scarce. The method is easy to perform, does not require laboratory infrastructure, can be performed by any health care provider after a short training, and provides immediate results [7] . Although a pilot study revealed improved screening uptake by using an HPV-DNA-based self-sampling method in Ethiopia [8], VIA is the recommended screening method in the country by the Federal Ministry of Health (FMOH) [7, 9]. Accordingly, more than 214 health facilities provided cervical cancer screening using the VIA screening method in 2019 [10]. However, screening coverage remains low and was only 2.9% in 2017 before all facilities offered service [11]. This progress did not fulfil the National Cancer Control Plan target to reach coverage of 80% by 2020 [7]. Previous screening uptake studies conducted in Ethiopia found that screening uptake was low [12–16]. A study in Addis Ababa found that self-reported screening uptake was 11.5% in 2015 [17], 10.8% in Addis Ababa in 2018 [16], 10% in Gondar in 2018 [13], and 20.9% in Debremarkos in 2017 [15], and 85.8% of women in Southern Ethiopia had no intention for screening at all [18].

In Ethiopia, the few studies that were conducted on cervical cancer screening uptake mainly focused on human immunodeficiency virus (HIV)-positive women in the hospital setting [13, 19]. Other uptake studies in Ethiopia were based on self-reported past screening experience [13–15, 19–21]. However, self-reported screening measurement was reported to possibly have limitations such as over-reporting [22–24]. To our knowledge, no study has measured screening uptake after actively inviting eligible women to VIA screening among eligible women at public health centres in Ethiopia. Therefore, in this study, we assessed the uptake of VIA after invitation to public health facilities and its associated socio-demographic factors in Gondar town, Northwest Ethiopia.

## Method

### Study design and setting

This facility-based, cross-sectional study was conducted at public health centres and a referral hospital in Gondar, Northwest Ethiopia. It was conducted among eligible women who visited the reproductive health services (family planning, immunisation, and under five children clinics) in four public health centres and Gondar Referral Hospital in Gondar town. Gondar city is located in the Amhara Regional State, 727 km north from Addis Ababa, the capital city of Ethiopia. Gondar has a total population of 206,987, and more than half (108,902) are females [25].

### Participants

Women aged 30–49 who sought reproductive health services and were advised/linked for cervical cancer screening in public health facilities in Gondar town were included in this study. Using a simple random sampling method, four health centres out of eight health centres (Teda HC, Azezo HC, Maraki HC, and Poli HC) were selected, and Gondar University Referral Hospital was selected purposively for the study.

Sample size was determined using a single population proportion formula with the assumptions of a 5% level of significance (95% confidence interval [CI]), a 4% margin of error (d), and a 24.8% (P) proportion of cervical cancer screening from a previously conducted facility-based study in Addis Ababa, Ethiopia [17]. After adjusting for a 10% non-response rate, the final sample size was calculated to be 493. The sample size was allocated proportionally to selected health facilities based on the previous two-month patient flow from data collection time. Then, a two-month active collection of data was performed. Based on eligibility criteria, women who met the eligibility criteria were consecutively approached by data collectors in the waiting area of reproductive health service OPD, informed consent was obtained, and women were included until the required sample size was achieved. Women were excluded if they had history of previous screening, pregnancy, active bleeding, and a previous hysterectomy.

We have used the standard screening method based on the national cervical cancer screening guideline. Demographic and other characteristics were collected by data collectors. The participants were then advised and linked to undergo VIA screening immediately after their visit to the health facility after brief information on cervical cancer and screening given by data collectors as per the WHO and Ministry of Health guidelines for screening. Women who refused the screening were asked their reason for refusal. The number of women who agreed to the screening was recorded, and the women were directed to the screening room. A unique identification number was given to participants. Then, the test result was checked to determine the actual uptake of screening.

### Variables and operational definitions

#### Cervical cancer screening uptake

Uptake was measured by the proportion of women who underwent the screening test after being advised/linked to the VIA test.

#### Knowledge

Knowledge of cervical cancer and screening was measured using 17 “yes or no” response-type items. A correct response was allocated a score of 1, otherwise 0. The scores were then summed, and women with a summary score greater than or equal to the mean score and women with a score less than the mean score were used to categorise good and poor knowledge, respectively [16].

#### Perception

Perceived benefit, perceived barrier, perceived susceptibility, perceived severity, cues to action, and self-efficacy for cervical cancer screening were measured based on five-point Likert scale items ranging from strongly disagree to strongly agree. There were three Likert scale items with a maximum score of 3 × 5 = 15 for perceived benefits, five Likert scale items with a maximum score of 5 × 5 = 25 for perceived barriers, and two Likert scale items with a maximum score of 2 × 5 = 10 for each of perceived susceptibility, severity, cues to action, and perceived self-efficacy constructs based on the health belief model (HBM). The likert scale items for each HBM construct were measured from strongly disagree (1 point) to strongly agree (5 points). The scores were then summed, and those who scored greater or equal to or less than the mean score were categorized into “high” or “low,” respectively, to the respective HBM constructs [26, 27].

The dependent variable was cervical cancer screening uptake. The independent variables were the socio-demographic characteristics (age group, education, occupation, religion, ethnicity, marital status, residency, income); knowledge about cervical cancer and cervical cancer screening; and perception of cervical cancer and screening based on the HBM, health facility, and accessibility conditions for cervical cancer screening service.

### Data sources/measurements

Quantitative data were collected using interviewer administered questionnaire. The questionnaire was adapted by reviewing relevant studies [13, 16, 28, 29]. The questions were written first in English and translated to the local language (Amharic) and then translated back into English to check for consistency. Five experienced data collectors were recruited and collected the data for 2 months after they received 3 days of training. The questionnaire was pre-tested on 5% of the calculated sample size among women age 30–49 years from two VIA health centres in Gondar town to assure the understandability of the tool. The reliability of the study instrument was ensured by calculating the Cronbach’s alpha coefficient to measure the internal consistency of knowledge and Likert scale items for HBM constructs. The Cronbach’s alpha coefficient was greater than 0.7 for all items.

### Methods of analysis

Data were entered using Epi Info version 7.2, and analysis was performed using SPSS version 25. Descriptive statistics (mean, median, standard deviation) and summary measures (frequency table and graphs) were used to summarise the variables under study. Independent variables were checked for multi-collinearity using variance inflation factor (VIF), and the correlations were acceptable. During analysis we have internally classified the study participants as receptors and refusals of the screening to see if they were different based on socio demographic characteristics and identify factors associated. Bivariate and multivariable logistic regression analyses were used to determine factors of cervical cancer screening uptake (*p*-value < 0.2 was used to select candidate variables for multivariable logistic regression). The regression model was first examined for goodness of fit test using the Hosmer–Lemeshow test, and the fitted model adequately described the data. Crude odds ratio (COR) and adjusted odds ratio (AOR) with a 95% CI were computed along with the corresponding *p*-value (*p* < 0.05) to determine statistical significance with the dependent variable.

## Results

### Socio-demographic characteristics of the respondents

Out of 493 women approached to participate in the study, 467 agreed to participate, yielding a response rate of 94.7%. The analyses were limited to 464 women with questionnaires that had complete information. The median age of respondents was 35 years, ranging from a minimum of 30 to a maximum of 49 years. The majority of 223 (48.1%) were in the age group of 30–34 years. More than two-thirds of respondents, 374 (80.6), were urban dwellers (Table 1).

**Table 1:** Distribution of screened and non-screened women according to socio-demographic characteristics in Gondar, Ethiopia, in 2019 (*N* = 464)

Variables		Total *n* = 464 (%)	Cervical cancer screening uptake	
			Non-uptake *n* = 388 (83.6%)	Uptake *n* = 76 (16.4%)
Age group	30–34	223 (48.1%)	187 (48.2%)	36 (47.4%)
	35–39	150 (32.3%)	134 (34.5%)	16 (21%)
	40–44	55 (11.9%)	42 (10.8%)	13 (17.1%)
	45–49	36 (7.7%)	25 (6.5%)	11 (14.5%)
Marital status	Single	10 (2.2%)	8 (2.1%)	2 (2.6%)
	Married	402 (86.6%)	330 (85.1%)	64 (84.2%)
	Divorced	34 (7.3%)	32 (8.2%)	7 (9.2%)
	Widowed	11 (2.4%)	13 (3.4%)	1 (1.3%)
	Separated	7 (1.5%)	5 (1.3%)	2 (2.6%)
Residence	Rural	90 (19.4%)	78 (20.1%)	12 (15.8%)
	Urban	374 (80.6%)	310 (79.9%)	64 (84.2%)
Education	No education	310 (66.8%)	277 (71.4%)	33 (43.4%)
	Primary & above	154 (33.2%)	111 (28.6%)	43 (56.6%)
Occupational status	Housewife	269 (58%)	230 (59.3%)	39 (51.3%)
	Government worker	44 (9.5%)	36 (9.3%)	8 (10.5%)
	Private job	151 (32.5%)	122 (31.4%)	29 (38.2%)
Income status	< 700	44 (9.5%)	35 (9%)	9 (11.8%)
	700–1400	182 (39.2%)	158 (40.7%)	24 (31.6%)
	1401–2100	90 (19.4%)	75 (19.3%)	15 (19.7%)
	2101–2800	50 (10.8%)	41 (10.6%)	9 (11.8%)
	> 2800	98 (21.1%)	79 (20.4%)	19 (25%)
Number of children	1 child	75 (16.2%)	64 (16.5%)	11 (14.5%)
	2–4 children	294 (63.4%)	240 (61.9%)	54 (71%)
	> 4 children	95 (20.5%)	84 (21.6%)	11 (14.5%)
Number of sexual partners	One	299 (64.4%)	238 (61.3%)	61 (80.2%)
	Two	153 (33%)	142 (36.6%)	11 (14.5%)
	More than three	12 (2.6%)	8 (2.1%)	4 (5.3%)
HIV status	Negative	448 (96.6%)	374 (96.4%)	74 (97.4%)
	Positive	5 (1%)	3 (0.8%)	2 (2.6%)
	do not know	11 (2.4%)	11 (2.8%)	0 (0%)

### Cervical cancer screening uptake

**Fig. 1:**
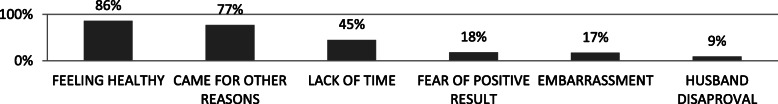
Reason for rejecting cervical cancer screening offer in Gondar, Ethiopia, in 2019

Three hundred eighty-eight (83.6%) participants did not opt to participate in cervical cancer screening tests after invitation. Only 76 (16.4%) of the study participants underwent the screening test. Most of the screened participants (70, 92.1%), had a negative VIA result. Only four (5.2%) were VIA-positive and were eligible for cryotherapy and treated accordingly. Two (2.6%) were suspicious for cancer and referred for further diagnosis and treatment. The most common reason mentioned for rejecting the screening test by study participants was the feeling of being healthy (334, 86%), followed by having come for another health service (297, 77%) (Fig. 1).

### Factors associated with cervical cancer screening uptake

In the multivariable logistic regression model, the women’s educational status, perceived susceptibility, perceived severity, perceived self-efficacy, cues to action, knowledge status, and previous recommendation by a health worker for screening were significantly associated with cervical cancer screening uptake (Table 2).

**Table 2:** Multivariable logistic regression analysis for factors associated with screening uptake in Gondar, Ethiopia, in 2019 (*N* = 464)

Variables		Uptake n (%)		COR (95% CI)	*p*-value	AOR (95% CI)	*p*-value
		No	Yes				
Age group	30–34	187 (48.2)	36 (47.4)	1	1	1	1
	35–39	134 (34.5)	16 (21)	0.62 (0.33,1.16)	0.13	0.9 (0.29, 0.77)	0.86
	40–44	42 (10.8)	13 (17.1)	1.6 (0.78, 3.29)	0.19	4.0 (1.15,14.07)	0.03
	45–49	25 (6.5)	11 (14.5)	2.2 (1.03, 5.05)	0.04	3.5 (0.85,14.40)	0.08
Education	No education	277 (71.4)	33 (43.4)	1	1	1	1
	Primary & above	111 (28.6)	43 (56.6)	3.2 (1.96, 5.38)	< 0.001	5.3 (2.20, 13.0)	< 0.001
Perceived benefit	Low	99 (25.5)	11 (14.5)	1	1	1	1
	High	289 (74.5)	65 (85.5)	2.0 (1.02, 3.99)	0.04	0.57 (0.20,1.61)	0.29
Perceived susceptibility	Low	344 (88.7)	19 (25)	1	1	1	1
	High	44 (11.3)	57 (75)	23.4 (12.7,43.0)	< 0.001	6.5 (2.72,15.51)	< 0.001
Perceived barrier	Low	225 (57.9)	10 (13.2)	9.1 (4.54,18.25)	< 0.001	6.4 (2.30,17.80)	< 0.001
	High	163 (42.1)	66 (86.8)	1	1	1	1
Perceived severity	Low	98 (25.3)	6 (7.9)	1	1	1	1
	High	290 (74.7)	70 (92.1)	3.9 (1.66, 9.36)	0.002	3.4 (1.01,11.65)	0.04
Cues to action	Low	333 (85.8)	23 (30.3)	1	1	1	1
	High	55 (14.2)	53 (69.7)	25.8 (13.9,47.8)	< 0.001	4.5 (1.86, 11.3)	0.001
Perceived self-efficacy	Low	308 (79.4)	26 (34.2)	1	1	1	1
	High	80 (20.6)	50 (65.8)	12 (7.07, 21.83)	< 0.001	5 (2.16,11.6)	< 0.001
Knowledge status	Low	350 (90.2)	33 (43.4)	1	1	1	1
	High	38 (9.8)	43 (56.6)	12 (6.83, 21.03)	< 0.001	8.4 (3.33,21.21)	< 0.001
Previously told about screening	No	260 (67)	34 (44.7)	1	1	1	1
	Yes	128 (33)	42 (55.3)	2.5 (1.52, 4.13)	< 0.001	2.7 (1.15, 6.51)	0.02
Knew the screening site	No	328 (84.5)	56 (73.7)	1	1	1	1
	Yes	60 (15.5)	20 (26.3)	1.9 (1.09, 3.48)	0.02	1.1 (0.39, 3.03)	0.85

NB: *COR* crude odds ratio, *AOR* adjusted odds ratio, *CI* confidence interval

Women who had more education were more likely to undergo cervical cancer screening than women without education (AOR = 5.3, 95% CI [2.20, 13.0]). Women who perceived a high susceptibility to cervical cancer were more likely to undergo screening than women who had low perceived susceptibility (AOR = 6.5, 95% CI [2.72, 15.51]). Women who had low perceived barriers were more likely to undergo cervical cancer screening than women with high perceived barriers (AOR = 6.4, 95% CI [2.30, 17.80]). Those mentioning high perceived severity of cervical cancer were more likely to undergo screening than those who had low perceived severity (AOR = 3.4, 95% CI [1.01, 11.85]). Women who had high cues to action were more likely to undergo cervical cancer screening than women who had low cues to action (AOR = 4.5, 95% CI [1.86, 11.32]). Those with high self-efficacy were more likely to undergo screening uptake than those who had low self-efficacy (AOR = 5, 95% CI [2.14, 11.73]). Those who had good knowledge regarding cervical cancer and screening were more likely to undergo the screening test (AOR = 8.4, 95% CI [3.33, 21.21]). Finally, women who had been recommended cervical cancer screening before were more likely to undergo cervical cancer screening (AOR = 2.7, 95% CI [1.15, 6.51]).

## Discussion

Cervical cancer screening uptake among women who visited Gondar public health facilities was one in eight after a personal invitation. Feeling healthy was the most common reason for VIA screening test refusal. Higher educational status, previous recommendation by health providers, higher knowledge status, higher perceived susceptibility, lower perceived barrier, higher perceived severity, shorter cues to action, and higher perceived self-efficacy were significantly associated with actual screening uptake.

This study found higher cervical cancer screening uptake by study participants (16.4%) compared to the national cervical cancer screening coverage (2.9%) [11]. Also, the study finding was consistent with other studies (no uptake after invitation), in which cervical cancer screening uptake was 16.4% among women in a survey conducted in Kenya in 2018 [30] and 17.5% among women seeking services at a health facility Kisumu, Kenya, in 2015 [31].

Although cervical cancer screening uptake in this study was not that high, it was higher than the previous uptake study of 10% in Gondar Hospital in 2018 without invitation [13], 10.6% in Addis Ababa in 2018 [16], 8% in Nigeria in 2016 [32], 6% in Kenya in 2013 [33], and 4.8% in Uganda in 2016 [34]. This small difference might be due to differences in population characteristics and study settings or facilities. The uptake report from these studies was not after invitation; it was based on retrospective assessment, and some studies were from an HIV clinic alone. For instance, the previously conducted study in Gondar focused on HIV-positive women who visited the referral hospital compared to the current study, which included women irrespective of HIV status attending health facilities and hospitals together (possible now due to service availability at lower-level facilities). Thus, increased uptake observed in the current study might be because of recent placement of VIA screening to the health centre level in the town, which is closer to the participants than where it had been only at the hospital level. Also, eligible women were actively invited and offered VIA screening in the current study compared to the previous studies. This might have helped women to easily identify and access the service and utilise it accordingly. Current cervical cancer prevention promotion programmes are spread throughout the country and there are sensitisation implementation activities, which may have led to higher uptake in our study.

However, the current finding is lower than the self-reported lifetime screening uptake of 23.4% among women in the Gondar University Referral Hospital in 2017 [19], 20.9% in Debre Markos, Ethiopia, in 2017 [15], 46% in Kenya in 2017 [35], 40.7% in Bahrain in 2018 [36], and 26% in Saudi Arabia in 2019 [37]. The difference might be due to a difference in the way cervical cancer screening uptake was measured. All the above-mentioned studies used lifetime self-reported experience as a measure for screening uptake [22–24] compared with uptake being measured at one time point in the current study.

The most common reasons for cervical cancer screening refusal were feeling healthy (86%), coming to visit the facility for other reasons (77.5%), lack of time (45%), fear of the test result (18%), and embarrassment (17%). This was consistent with the study done in Butajira, Ethiopia, documenting a lack of time to undergo screening, self-assertion of being healthy, and fear of screening as the main reasons for cervical cancer screening test refusal [10]. This highlights that women might not undergo the screening service simply because they do not understand why the screening test is needed and who is eligible for the service. Therefore, screening programmes should consider potentially hindering factors for screening uptake.

This study found that among socio-demographic characteristics, educational status had an independent association with cervical cancer screening uptake. This is consistent with studies in Gondar where higher education level also increased the odds of uptake [13]; similar findings were reported in Kenya [31]. This means that women who are not educated have limited access to cervical cancer screening programmes [18]. To increase cervical cancer screening uptake, better ways to attract women without formal education have to be found. In the multivariable model, there was no independent statistical association between other socio-demographic factors and uptake of screening. For instance, we did not find statistically significant association between income factor and uptake. This might be due to the fact that the screening test is available without fee in the health facilities and did not influence the uptake rate in our study.

In this study, the history of previous recommendation by a health professional for screening was significantly associated with cervical cancer screening uptake. This is consistent with findings from Uganda [34]. This implies that to increase uptake of cervical cancer screening by eligible women, health workers can recommend screening to eligible women whenever they encounter them at health facilities. This may have a lagged effect, even if the women do not immediately adhere to the recommendation.

Knowledge status among study participants (382, 82.3%) was poor in Gondar public health facilities based on the mean score computed from 17 items assessing the risk factors, symptoms, treatment, and prevention methods of cervical cancer used by other studies, and it had a strong independent statistical association with cervical cancer screening uptake (AOR = 8.4, 95% CI [3.33, 21.21]). This is consistent with studies in developing countries in general where knowledge of cervical cancer and screening is poor. A similar finding was reported in Gondar Hospital that comprehensive knowledge was poor (79.8%) and was a strong predictor variable for screening uptake in that study (AOR = 3.02, 95% CI [2.31, 7.15]) [19]. The finding is also in line with a systematic review and meta-analysis conducted among HIV-positive women in Ethiopia in 2020 that showed women who had good knowledge of cervical cancer were more likely to be screened for cervical cancer than their counterparts [38]. This suggested that the higher the knowledge of women about the screening program, the more their participation which underscores the need to improve their comprehensive knowledge about cervical cancer and cervical cancer screening to increase their screening uptake.

The perception of the women based on HBM constructs regarding cervical cancer and screening was found to have an impact on cervical cancer screening uptake. In this study, perceived higher susceptibility of cervical cancer was statistically associated with cervical cancer screening uptake (AOR = 6.5, 95% CI [2.72, 15.51]). This finding is similar to a study in Mekelle, Ethiopia, in 2015 [21], in which perceived susceptibility was significantly associated with lifetime cervical cancer screening uptake (AOR = 2.2, 95% CI [1.30, 3.78]). It is also similar to a study in Botswana in 2017 [39], in which perceived susceptibility was significantly associated with screening uptake (AOR = 1.8, 95% CI [1.094–3.067]). This could be due to women who undergo cervical cancer screening test believed that they are at risk of cervical cancer. Conversely, feeling of healthy was the commonest reason for rejecting the screening test (86% in the current study). Therefore educational campaigns should consider increasing women perceived susceptibility.

About half of the participants in this study had a low perceived barrier (235, 50.6%). In multivariable logistic regression, a low perceived barrier by study participants had a strong statistical association with cervical cancer screening uptake (AOR = 6.4, 95% CI [2.30, 17.80]). This is in line with a 2015 study in Mekelle, Ethiopia, in which perceived barriers were significantly associated with cervical cancer screening uptake (AOR = 2.256, 95% CI [1.447–3.517]) [21]. It is important, therefore, to remove perceived barriers, such as the belief that cervical cancer screening would be painful and embarrassing.

This study also revealed that perceived severity of cervical cancer among study participants in Gondar town public health facilities was common (76.6%) and significantly associated with cervical cancer screening uptake (AOR = 3.4, 95% CI [1.019, 11.851]). This is consistent with a study in Kenya in which those who perceived cervical cancer as a serious disease had higher screening uptake than those who did not [31]. This implies that although more women perceived cervical cancer as severe, few of them utilised the screening service. Therefore, programmes to explain severity will not change much, since many women already believe that cervical cancer is severe. This highlights the need to consider additional ways that can have immediate effect on the decision to accept the service. Furthermore, fear about cervical cancer and cervical cancer screening might lead women to avoid screening. Therefore reassuring women with high perceived severity to overcome their fear might play a positive role to improve screening uptake after invitation. Women might also decline to participate due to perceiving themselves healthy, not having enough time, fear of bad news from others, feeling of sham about the screening [10]. This in turn underscores the need to address these factors as well in cervical cancer screening programs.

This study also revealed cues to action were also significantly associated with cervical cancer screening uptake. This is consistent with a study in Kenya in 2014 [31]. Similarly other studies revealed that physician advice for cervical cancer screening [40], someone they knew screened before, information from mass media and educational talks were important cues for women to attend the screening test [41]. This finding implies the need to consider important cues to take action in cervical cancer screening programs to improve the screening uptake by women. In terms of self-efficacy, one in four study participants had high self-efficacy regarding cervical cancer screening and had a strong statistical association with cervical cancer screening uptake. This is consistent with a study in Nigeria that reported that the confidence of women regarding screening resulted in increased screening uptake [42]. It is therefore necessary to educate and empower women as often as possible to boost their confidence. Doing so will help them to make decisions to utilise the already freely available cervical cancer screening service in health facilities.

### Strengths and limitations

This study reflects a real-life practice and effect of the “see and treat” approach currently implemented by the Ethiopian government: women visiting a health centre for routine services were invited and uptake was measured. We postulate that this magnitude of uptake during a single visit is representative for the strategy without additional campaigns or incentives. There is no clear uptake target for this situation, and the impact on the overall screening uptake over 1 year is not clear. Additional sensitisation over some time may still increase the uptake. Since the study was facility-based and used consecutive sampling techniques to select the study participants in Gondar, generalisability to the whole of Ethiopia cannot be applied.

## Conclusion

This study found reasonable uptake after a brief invitation of age-eligible cervical cancer screening in Gondar public health facilities. The Ethiopian Ministry of Health (EMOH) in collaboration with regional, zonal, and Gondar town health administrators should a) encourage development of tailored messages to the local context, especially for women with little formal education, and b) formally link information about the service to family planning and under-five services, since at least some uptake can be achieved. Furthermore, health workers in health facilities should advise and motivate women to undergo screening tests whenever possible to increase the chance of future utilisation. Population-based screening registries at sentinel sites could monitor screening uptake over time and the effects of campaigns at individual health facilities and in the public.

## Data Availability

The datasets used during the study are available from the corresponding author upon reasonable request.

## Abbreviations

AOR: Adjusted odds ratio
CCa: Cervical cancer
CI: Confidence interval
COR: Crude odds ratio
EMOH: Ethiopian Ministry of Health
HBM: Health belief model
SD: Standard deviation
SAT: Sea-and-treat approach
VIA: Visual inspection with acidic acid
WHO: World Health Organisation
